# Gene Expression of *IGF1*, *IGF1R*, and *IGFBP3* in Epiretinal Membranes of Patients with Proliferative Diabetic Retinopathy: Preliminary Study

**DOI:** 10.1155/2013/986217

**Published:** 2013-11-28

**Authors:** Dorota Romaniuk, Malgorzata W. Kimsa, Barbara Strzalka-Mrozik, Magdalena C. Kimsa, Adam Kabiesz, Wanda Romaniuk, Urszula Mazurek

**Affiliations:** ^1^Clinical Department of Ophthalmology, Medical University of Silesia, Ceglana Street 35, 40-952 Katowice, Poland; ^2^Department of Molecular Biology, Medical University of Silesia, Narcyzów Street 1, 41-200 Sosnowiec, Poland

## Abstract

The molecular mechanism formation of secondary epiretinal membranes (ERMs) after proliferative diabetic retinopathy (PDR) or primary idiopathic ERMs is still poorly understood. Therefore, the present study focused on the assessment of *IGF1*, *IGF1R*, and *IGFBP3* mRNA levels in ERMs and PBMCs from patients with PDR. The examined group comprised 6 patients with secondary ERMs after PDR and the control group consisted of 11 patients with idiopathic ERMs. Quantification of *IGF1*, *IGF1R*, and *IGFBP3* mRNAs was performed by real-time QRT-PCR technique. In ERMs, *IGF1* and *IGF1R* mRNA levels were significantly higher in patients with diabetes compared to control subjects. In PBMCs, there were no statistically significant differences of *IGF1*, *IGF1R*, and *IGFBP3* expression between diabetic and nondiabetic patients. In conclusion, our study indicated *IGF1* and *IGF1R* differential expression in ERMs, but not in PBMCs, of diabetic and nondiabetic patients, suggesting that these factors can be involved in the pathogenesis or progression of proliferative vitreoretinal disorders. This trial is registered with NCT00841334.

## 1. Introduction

Proliferative diabetic retinopathy (PDR) is a common and specific microvascular complication of diabetes mellitus. Inflammation, angiogenesis, and fibrosis are primary processes involved in the pathogenesis of PDR [1]. Moreover, Snead et al. [2] suggested that hypoxia and neovascular cytokine production may play an important role in epiretinal membrane (ERM) formation of PDR patients.

Epiretinal membrane is a layer of pathologic tissue which grows on the internal surface of the retina [3, 4]. ERMs can be idiopathic (iERM) or secondary to some pathologic conditions, including proliferative diabetic retinopathy, proliferative vitreoretinopathy (PVR), macular pucker, high myopia, uveitis, and other retinal degenerative diseases [5]. Depending on duration and severity of ERMs, the symptoms felt by patient can differ from mild to severe metamorphopsia, decrease in visual acuity caused by retinal distortion and problems such as vitreoretinal tractions or even tractional retinal detachment [6]. It is known that ERMs in PDR are mainly composed of new pathological vessels. On the contrary, the idiopathic ERMs consist of a nonangiogenic fibroglial tissue [7]. However, the molecular mechanism formation of both the primary and secondary ERMs in diabetic and nondiabetic patients is still poorly understood.

Several cytokines, including insulin-like growth factor (IGF), are involved in the progression of ERMs [8–10]. The IGF family is comprised of ligands: IGF1, IGF2, insulin; six binding proteins (IGFBP1 through -6); and cell surface receptors, including IGF1R, IGF2R, and insulin receptors [11]. Many recent studies suggested that IGF1 and IGF2 may be involved in the pathogenesis of PDR; however, the exact role has not been well understood yet [12, 13]. Recent reports suggest that increases in IGF1 activity may contribute to retinal neovascularization characteristic for PDR [14, 15]. IGFs are also involved in stimulation of epiretinal membrane contraction [9]. Additionally, IGF system is known to be associated with inflammation process [16, 17].

Numerous attempts have been made to identify various proteins in serum or mRNA in peripheral blood mononuclear cells (PBMCs), which could be easily accessed and act as markers of intratissue processes in various diseases [13, 18]. However, local concentrations of different growth factors in the retina may be more important than systemic levels in the pathogenesis of diabetic retinopathy. There are only few published data regarding differences between mRNA levels of *IGF* in the eye tissues of diabetic and nondiabetic patients [19–22]. Therefore, the present study focused on the assessment of mRNA levels of *IGF1*, *IGF1R*, and *IGFBP3* both in ERMs and PBMCs from patients with proliferative diabetic retinopathy.

## 2. Materials and Methods

### 2.1. Subjects

The blood samples from 17 patients and surgically removed ERMs from 17 eyes of these patients were obtained and categorized into diabetic and nondiabetic ones. The examined group comprised 6 patients (3 females and 3 males, mean age 72.9 years; range 66–80 years) with secondary ERMs after PDR and the control group consisted of 11 patients (8 females and 3 males, mean age 69.7; range 61–79 years) with iERMs (Table 1). All the patients were treated in the Department of Ophthalmology, University Hospital No. 5, Medical University of Silesia, Katowice, Poland.

The period between the diagnosis of the condition and the actual surgery for the condition was between 2 and 16 months. Some patients presented and had surgery within two month. When patients were asymptomatic or had good visual acuity ERMs were diagnosed clinically that did not require immediate surgery. The average duration in which the membranes were present in the eye before removal was 6.4 months (range 3–10 months) for PDR and 8.6 months (range 2–16 months) for iERM. All subjects underwent a complete ophthalmic examination: best corrected visual acuity (BCVA), tonometry, slit lamp examination of anterior and posterior segment, ultrasonography of the eye, and optical coherence tomography (OCT) of the central retina. The criteria for inclusion into the molecular analysis were as follows: patients of Caucasian race, both males and females, aged ≥55 years, suffering from idiopathic ERMs or secondary ERMs in PDR. Presence of ERM was proven in OCT examination. All patients were qualified to surgical treatment of ERM—unilateral pars plana vitrectomy (PPV).

The study was approved by the Bioethics Committee of the Medical University in Katowice (KNW/0022/KB1/82/12) in accordance with the Declaration of Helsinki regarding medical research involving human subjects. All patients were informed about the research and signed an informed consent form.

### 2.2. Tissues

Samples of all ERMs were collected during pars plana vitrectomy, using intraocular microforceps, and were stored for 48 h at −70°C until RNA extraction. Venous blood samples were collected into EDTA-containing tubes and peripheral mononuclear cells were isolated from 5 mL specimens derived from each patients by using Ficoll-Conray density gradient centrifugation for 30 min at 1500 rpm at room temperature immediately after blood collection (specific gravity 1.077; Immunobiological Co., Gunma, Japan).

### 2.3. Ribonucleic Acid Extraction from Tissue Specimens

Total RNA was extracted from ERMs and PBMCs using a TRIzol reagent (Invitrogen, Carlsbad, CA, USA). RNA extracts were treated with DNase I (MBI Fermentas, Vilnius, Lithuania) according to the manufacturer's instructions. The quality of extracts was checked electrophoretically using 0.8% agarose gel stained with ethidium bromide. The results were analyzed and recorded using the 1D Bas-Sys gel documentation system (Biotech-Fisher, Perth, Australia). Total RNA concentration was determined by spectrophotometric measurement in 5 *μ*L capillary tubes using the Gene Quant II RNA/DNA Calculator (Pharmacia Biotech, Cambridge, UK).

### 2.4. Quantitative Real-Time Polymerase Chain Reaction Assay

Detection of the expression of *IGF1*,* IGF1R*, *IGFBP3*, and glyceraldehyde-3-phosphate (*GAPDH*) mRNAs was carried out using a QRT-PCR technique with SYBR Green chemistry (SYBR Green Quantitect RT-PCR Kit, Qiagen) and an Opticon DNA Engine Continuous Fluorescence detector (MJ Research) as described previously [23]. All samples were tested in triplicate. *GAPDH* for each sample was measured to exclude possible RT-PCR inhibitors. Oligonucleotide primers, specific for *IGF1*, *IGF1R*, and *IGFBP3*, were designed on the basis of reference sequences (GenBank accession no. NM_000618.3, NM_000875.3, and NM_000598.4, resp.) using Primer ExpressTM Version 2.0 software (PE Applied Biosystems, Foster City, CA, USA). Detection of *GAPDH* mRNAs was performed as described previously [23] (Table 2). The thermal profile for one-step RT-PCR was as follows: reverse transcription at 50°C for 30 min, denaturation at 95°C for 15 min, and 40 cycles consisting of the following temperatures and time intervals: 94°C for 15 s, 60°C for *GAPDH*, 61.1°C for *IGF1*, 56.4°C for *IGF1R* and 54.2°C for *IGFBP3* for 30 s, and 72°C for 30 s. Each run was completed using melting curve analysis to confirm the specificity of amplification and the absence of primer dimers. RT-PCR products were separated on 6% polyacrylamide gels and visualized with silver salts.

### 2.5. Quantification of Expression of Target Genes

To quantify the results obtained with RT-PCR for *IGF1*, *IGF1R*, *IGFBP3*, and *GAPDH*, a standard curve method was used [23, 24]. Commercially available standards of *β*-actin cDNA (TaqMan DNA Template Reagent Kit, PE Applied Biosystems, Foster, CA) were used at five different concentrations (0.6, 1.2, 3.0, 6.0, and 12.0 ng/*μ*L), to simultaneously detect the expression profile of each investigated gene. For standards, the calculation of copy number values was based on the following relationship: 1 ng of DNA = 333 genome equivalents (PE Applied Biosystems). Amplification plots for each dilution of a commercially available standard template were used to determine the Ct values. A standard curve was generated by plotting the Ct values against the log of the known amount of *β*-actin cDNA copy numbers. Correlation coefficients for standard curves ranged from 0.988 to 0.995 indicating a high degree of confidence for measurement of the copy number of molecules in each sample.

### 2.6. Statistical Analyses

Statistical analyses were performed using Statistica 9.0 software (StatSoft, Tulsa, OK, USA), and the level of significance was set at *P* < 0.05. Values were expressed as median with the 25th and 75th quartiles, and minimum and maximum. The Mann-Whitney *U*-test was applied to assess differences in the expression level of *IGF1*,* IGF1R*, and *IGFBP3*. Correlations were evaluated using the Spearman rank correlation test.

## 3. Results

### 3.1. Specificity of the Real-Time Reverse Transcription Polymerase Chain Reaction Assay

RT-PCR specificity for the target genes was confirmed experimentally on the basis of amplimers melting temperatures. For each RT-PCR product, a single peak at expected temperatures was observed: *IGF1*, 77.6°C; *IGF1R*, 77.4°C; *IGFBP3*, 82.4°C, and *GAPDH*, 80.0°C (data not shown). Gel electrophoresis also revealed the presence of a single product of predicted length (data not shown).

### 3.2. Differences in *IGF1*, *IGF1R*, and *IGFBP3* Expression Level between Diabetic and Nondiabetic Patients

**Figure 1:**
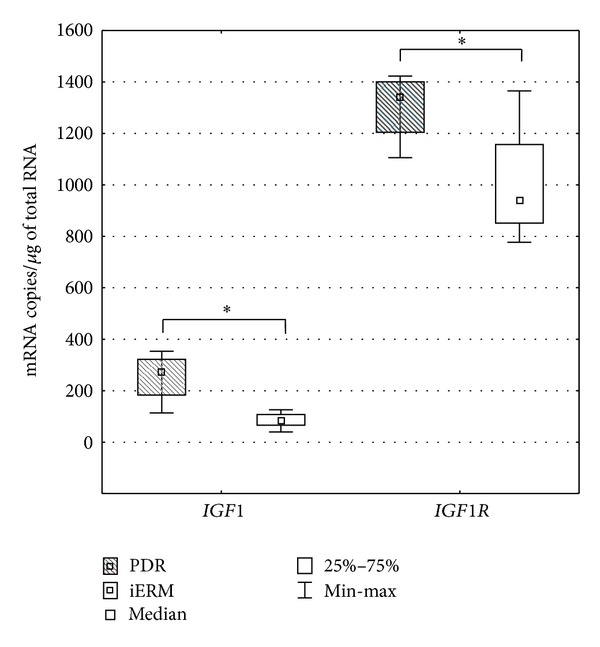
The mRNA levels of* IGF1* and *IGF1R* in epiretinal membranes of patients with proliferative diabetic retinopathy (PDR) and in the control subjects with idiopathic ERMs (iERM). (Box and whisker plots present medians  ±  quartiles and extreme values of copy numbers per 1 *μ*g of total RNA, statistical significance: *P < 0.05, Mann-Whitney *U*-test).

*IGF1* and *IGF1R* mRNAs were detected in all tested ERMs and PBMCs obtained from the control and study groups. However, *IGFBP3 *was only detected in PBMCs of all patients. In the ERMs, the mRNA level of *IGF1* was significantly higher in the patients with diabetes (median = 271 mRNA copies/*μ*g of total RNA) compared to the control iERMs of nondiabetic group (median = 85 mRNA copies/*μ*g of total RNA). In the case of *IGF1R*, the expression was significantly greater in ERMs after PDR (median = 1341 mRNA copies/*μ*g of total RNA) compared to the idiopathic ERMs (median = 938 mRNA copies/*μ*g of total RNA) (*P* = 0.0224, Mann-Whitney *U*-test) (Figure 1).

**Figure 2:**
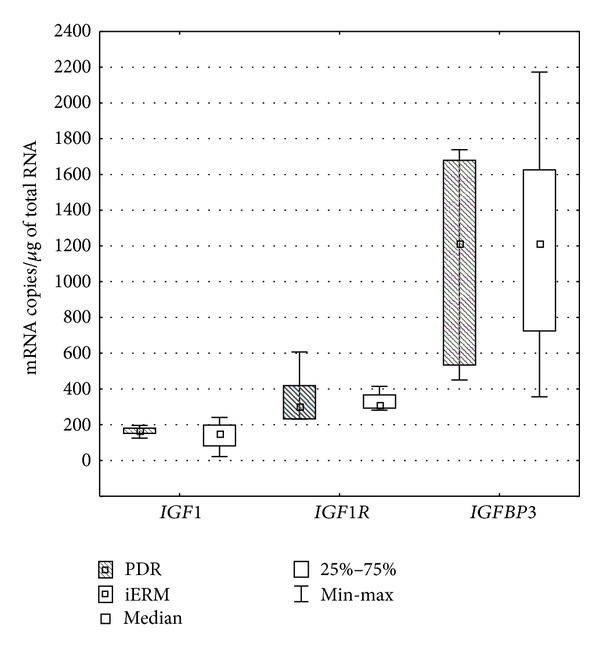
The mRNA levels of* IGF1*, *IGF1R*, and *IGFBP3* in peripheral blood mononuclear cells of patients with proliferative diabetic retinopathy (PDR) and in the control subjects with idiopathic ERMs (iERM). (Box and whisker plots present medians ± quartiles and extreme values of copy numbers per 1 *μ*g of total RNA, statistical significance: *P < 0.05, Mann-Whitney *U*-test).

In PBMCs, there were no significantly different expressions of *IGF1*, *IGF1R*, and *IGFBP3 *between diabetic (median = 163 mRNA copies/*μ*g of total RNA; 298 mRNA copies/*μ*g of total RNA; 1211 mRNA copies/*μ*g of total RNA, resp.) and nondiabetic patients (median = 144 mRNA copies/*μ*g of total RNA; 308 mRNA copies/*μ*g of total RNA; 1209 mRNA copies/*μ*g of total RNA, resp.) (*IGF1 P* = 0.6353, *IGF1R* *P* = 0.7250, *IGFBP3* *P* = 0.9599, Mann-Whitney *U*-test) (Figure 2).

### 3.3. Correlations between *IGF1*, *IGF1R*, and *IGFBP3* Expression Level and Time Interval between Diagnosis and Surgery

There was no correlation between the time interval between diagnosis of the condition and the actual surgery and the expression of *IGF1*, *IGF1R*, and *IGFBP3* both in the ERMs and PBMCs of the diabetic samples. Corresponding to the results obtained for diabetic patients, in iERMs and PBMCs from the nondiabetic patients, no correlations between the expression of *IGF1*, *IGF1R*, and *IGFBP3 *genes and time interval between diagnosis of the condition and the actual surgery were detected (Table 3).

## 4. Discussion

IGF1/IGF1R/IGFBPs complex can participate in the physiological and pathological events that occur in the retina. Previous research has revealed *IGF1*, *IGF1R*, and *IGFBPs* expression in microvascular endothelial cells, pericytes and retinal epithelial cells (RPE) *in vitro* [25–27]. Therefore, it is suggested that these growth factors can also cause progression of ERMs, especially during proliferative diabetic retinopathy [11]. On the other hand, the level of IGFs in the eye tissues may result, in part, from damage to the blood-retinal barrier. Furthermore, the association between level of IGFs in serum/PBMCs and the development of PDR is still not fully explained [12]. Therefore, determination of differences between mRNA levels of IGFs in ERMs and PBMCs in diabetic compared to nondiabetic patients using real-time QRT-PCR seems to be important. In the present study, *IGF1*, *IGF1R*, and *IGFBP3* mRNA levels were detected in PBMC samples, but in ERMs only *IGF1* and *IGF1R* mRNAs were determined. It can be explained that IGFBP3 did not cause proliferation of retinal cells [28]. Moreover, Spoerri et al. [29] showed that exogenous administration of IGFBP3 can induce growth inhibition and apoptosis of retinal cells. On the other hand, it was reported that IGFBP3 is neuroprotective, antiapoptotic, anti-inflammatory after retinal injury [30, 31] and may prevent the progression of PDR [32]. Previous research showed that also IGFs are not involved in proliferation of these cells but in stimulation of epiretinal membrane contraction [9]. In contrast, Spraul et al. [12] and Takagi et al. [25] indicate that IGF1 and IGF2 can influence RPE cell migration and proliferation. In turn, Ruberte et al. [33] determined that neovascularization was consistent with increased vascular endothelial growth factor level induced by IGF1 in retinal cells.

Statistically significant higher mRNA levels of *IGF1* and *IGF1R* in the ERMs of patients with PDR compared to the control subjects with iERMs in our results confirmed that the IGFs may play a key role in development or progression of PDR. Similar results were demonstrated by other authors who found significantly higher protein level of IGF1 in PDR than in control patient but in human vitreous samples [34–36], whereas Gerhardinger et al. [37] reported a lower mRNA levels of *IGF1* in diabetic retinas obtained postmortem from human donors and they did not observe a changed expression of *IGF1R*.

Interestingly, our results for the quantitative analysis of *IGF1*, *IGF1R,* and *IGFBP3* in PBMCs revealed no statistically significant differences of all three genes between diabetic and nondiabetic patients which might suggest that the processes occurring in the eye tissues are not affected by systemic expression levels of these cytokines during diabetes. Corresponding to our results, Payne et al. [13] and Spranger et al. [36] found that diabetic subjects had similar IGF1 level compared to nondiabetic subjects but in serum samples. Similarly, other authors showed no correlation between serum IGF1 levels and the development or progression of PDR [13, 38]. On the other hand, several studies contradict these findings; for example, Dills et al. [39] and Merimee et al. [40] have revealed higher serum IGF1 levels in diabetic patients. Moreover, Chen et al. [14] suggested that serum IGF1 level may be a contributing factor in diabetic retinopathy. However, these differences may be a result of the assay method used to measure IGF1 levels. Additionally, transport mechanisms for IGFs and IGFBPs between the blood and the eye are not very well known and may depend on the blood-retinal barrier whose disruption is an important event in pathogenesis of diabetic retinopathy and other ocular disorders [12]. Haurigot's studies demonstrated that intraocular IGF1, but not systemic IGF1, is sufficient to trigger blood-retinal barrier breakdown [15]. It may suggest that treatment strategies should be designed to counteract local than systemic IGF1 effects [15]. In contrast, Spranger et al. [36] indicated that breakdown of the blood-retinal barrier and serum levels of IGF mainly determined intravitreal IGF levels rather than local production. The disruption of the blood-retinal barrier can cause also the infiltration of immunologically competent cells into PDR membranes; therefore, the inflammatory reactions may play a role in the development of the epiretinal membrane in PDR [41]. It is also known that IGF1 has anti-inflammatory effects and it can be negatively correlated with inflammatory markers [16]. No correlation between the vitreous and serum levels of various growth factors in patients with PDR and nonproliferative diabetic retinopathy was also observed [42–44]. The lack of correlations between serum and intraocular tissue levels can suggest that serum/PBMC levels of various growth factors may not be a useful predictor of the diabetic retinopathy severity [42, 43]. Moreover, our research did not show any correlation between *IGF1*, *IGF1R*, and *IGFBP3* mRNA levels and the time interval between diagnosis of the condition and the surgery.

In conclusion, our study indicated *IGF1* and *IGF1R* differential expression in ERMs, but not in PBMCs, of diabetic and nondiabetic patients, suggesting that these factors can be involved in the pathogenesis or progression of proliferative vitreoretinal disorders. Unfortunately, major limitation of our study is a relatively small number of samples. However, analysis of ocular samples is often difficult because of the technical obstacles associated with extracting sufficient quantities of the samples. Therefore, there is a need to study a larger population and carry out further analysis, for example, on protein levels, in order to clarify the contribution of these intravitreal growth factors in the development of diabetic and nondiabetic ERMs. Moreover, this will help to identify better treatment strategies for proliferative diabetic retinopathy.

## Figures and Tables

**Table 1:** Selected clinical features of patients with secondary ERMs after proliferative diabetic retinopathy and the control group with idiopathic ERMs.

Characteristic	Secondary ERMs group (*n* = 6)	Idiopathic ERMs group (*n* = 11)
Gender		
Female	3	8
Male	3	3
Age (years)	72.9 (66–80)*	69.7 (61–79)
Eye		
Right	2	5
Left	4	6
Mean time interval between ERM diagnosis and surgery (months)	6.4 (3–10)	8.6 (2–16)
Visual acuity^†^	0.3 (0.01–0.5)	0.2 (0.02–0.4)

*Values of clinical parameters are expressed as means (minimum–maximum).

^†^Snellen chart.

**Table 2:** Characteristics of primers used for real-time QRT-PCR.

Gene	Sequence of primers	Length of amplicon (bp)*	Tm (°C)^†^
*IGF1 *	Forward: 5′-TGCTTCCGGAGCTGTGATC-3′	221	77.6
	Reverse: 5′-GATCCTGCGGTGGCATGTCACTCTTCACT-3′		
*IGF1R *	Forward: 5′-ACGCCAATAAGTTCGTCCACAGAGACCT-3′	187	77.4
	Reverse: 5′-GAAGACTCCATCCTTGAGGGACTCAG-3′		
*IGFBP3 *	Forward: 5′-GCTACAGCATGCAGAGCAAGT-3′	102	82.4
	Reverse: 5′-CAGCTGCTGGTCATGTCCTT-3′		
*GAPDH *	Forward: 5′-GAAGGTGAAGGTCGGAGTC-3′	226	80.0
	Reverse: 5′-GAAGATGGTGATGGGATTC-3′		

^∗^bp: base pairs.

^†^Tm: melting temperature.

**Table 3:** The correlations between relative expression of *IGF1*, *IGF1R*, and *IGFBP3* and time interval between diagnosis and surgery in the ERMs and PBMCs of diabetic and nondiabetic patients.

Diabetic patients						
ERMs	*IGF1 *	*IGF1R *	PBMCs	*IGF1 *	*IGF1R *	*IGFBP3*
Time interval between diagnosis and surgery	*r* = 0.316	*r* = −0.316	Time interval between diagnosis and surgery	*r* = 0.462	*r* = 0.103	*r* = −0.462

Nondiabetic patients						
iERMs	*IGF1 *	*IGF1R *	PBMCs	*IGF1 *	*IGF1R *	*IGFBP3 *

Time interval between diagnosis and surgery	*r* = 0.065	*r* = 0.497	Time interval between diagnosis and surgery	*r* = 0.153	*r* = 0.470	*r* = −0.442

Statistical significance: *P < 0.05, spearman rank correlation test.
